# Bis(2,6-diamino­pyridinium) bis­(hydrogen oxalate) monohydrate

**DOI:** 10.1107/S1600536811004119

**Published:** 2011-02-09

**Authors:** Mohammad T. M. Al-Dajani, Jamal Talaat, Shaharum Shamsuddin, Madhukar Hemamalini, Hoong-Kun Fun

**Affiliations:** a School of Pharmaceutical Sciences, Universiti Sains Malaysia, 11800 USM, Penang, Malaysia; b Virginia Commonwealth University, Medicinal Chemistry, USA; c Kampus Kesihatan, Universiti Sains Malaysia, 16150 Kubang Kerian, Kelantan, Malaysia; d X-ray Crystallography Unit, School of Physics, Universiti Sains Malaysia, 11800 USM, Penang, Malaysia

## Abstract

The asymmetric unit of the title compound, 2C_5_H_8_N_3_^+^·2C_2_HO_4_^−^·H_2_O, contains two crystallographically independent 2,6-diamino­pyridinium cations, a pair of hydrogen oxalate anions and a water mol­ecule. Both 2,6-diamino­pyridinium cations are planar, with maximum deviations of 0.011 (2) and 0.015 (1) Å, and are protonated at the pyridine N atoms. The hydrogen oxalate anions adopt twisted conformations and the dihedral angles between the planes of their carboxyl groups are 31.01 (11) and 63.48 (11)°. In the crystal, the cations, anions and water mol­ecules are linked *via* O—H⋯O and N—H⋯O hydrogen bonds, forming a three-dimensional network.

## Related literature

For applications of 2,6-diamino­pyridine, see: Abu Zuhri & Cox (1989 ▶). For related structures, see; Schwalbe *et al.* (1987 ▶); Al-Dajani *et al.* (2009 ▶, 2010 ▶); Aghabozorg *et al.* (2005 ▶); Büyükgüngör & Odabaşoğlu (2006 ▶); Odabaşoğlu & Büyükgüngör (2006 ▶); Haddad & Al-Far (2003 ▶). For details of oxalic acid, see: Subha Nandhini *et al.* (2001 ▶); Bahadur *et al.* (2007 ▶); Athimoolam & Natarajan (2007 ▶). For hydrogen-bond motifs, see: Bernstein *et al.* (1995 ▶). 
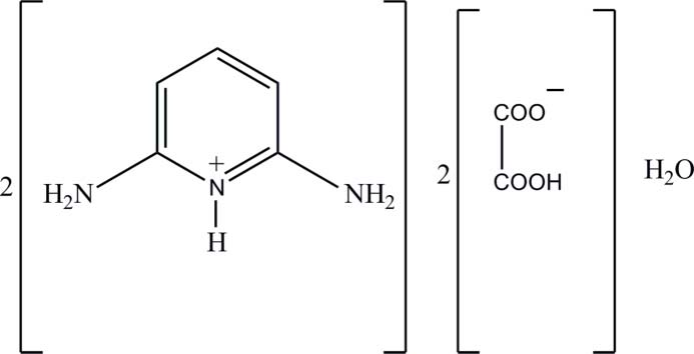


## Experimental

### 

#### Crystal data

2C_5_H_8_N_3_^+^·2C_2_HO_4_^−^·H_2_O
*M* *_r_* = 416.36
Monoclinic, 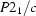

*a* = 8.1681 (1) Å
*b* = 34.8396 (4) Å
*c* = 7.2031 (1) Å
β = 114.573 (1)°
*V* = 1864.16 (4) Å^3^
*Z* = 4
Mo *K*α radiation
μ = 0.13 mm^−1^
*T* = 296 K
0.29 × 0.27 × 0.15 mm

#### Data collection

Bruker SMART APEXII CCD area-detector diffractometer
Absorption correction: multi-scan (*SADABS*; Bruker, 2009) ▶ *T*_min_ = 0.964, *T*_max_ = 0.982
33926 measured reflections
4287 independent reflections
3195 reflections with *I* > 2σ(*I*)
*R*_int_ = 0.037

#### Refinement

*R*[*F* ^2^ > 2σ(*F* ^2^)] = 0.048
*wR*(*F* ^2^) = 0.127
*S* = 1.03
4287 reflections
318 parameters
H atoms treated by a mixture of independent and constrained refinement
Δρ_max_ = 0.49 e Å^−3^
Δρ_min_ = −0.30 e Å^−3^

### 

Data collection: *APEX2* (Bruker, 2009 ▶); cell refinement: *SAINT* (Bruker, 2009 ▶); data reduction: *SAINT*; program(s) used to solve structure: *SHELXTL* (Sheldrick, 2008 ▶); program(s) used to refine structure: *SHELXTL*; molecular graphics: *SHELXTL*; software used to prepare material for publication: *SHELXTL* and *PLATON* (Spek, 2009 ▶).

## Supplementary Material

Crystal structure: contains datablocks global, I. DOI: 10.1107/S1600536811004119/yk2001sup1.cif

Structure factors: contains datablocks I. DOI: 10.1107/S1600536811004119/yk2001Isup2.hkl

Additional supplementary materials:  crystallographic information; 3D view; checkCIF report

## Figures and Tables

**Table 1:** Hydrogen-bond geometry (Å, °)

*D*—H⋯*A*	*D*—H	H⋯*A*	*D*⋯*A*	*D*—H⋯*A*
N1*A*—H1*NA*⋯O3*B*^i^	0.89 (2)	1.99 (2)	2.8668 (19)	172.5 (18)
O1*W*—H1*W*⋯O1*A*^ii^	0.82 (3)	2.53 (3)	3.219 (3)	142 (3)
O1*W*—H1*W*⋯O2*B*^ii^	0.82 (3)	2.28 (3)	2.984 (2)	145 (3)
N2*A*—H3*NA*⋯O4*B*	0.86 (3)	2.11 (3)	2.972 (3)	173.3 (18)
O2*A*—H2*A*⋯O3*B*^i^	0.94 (3)	1.65 (3)	2.5743 (18)	169 (3)
O2*B*—H2*B*⋯O3*A*	0.95 (3)	1.58 (3)	2.525 (2)	172 (3)
O1*W*—H2*W*⋯O1*B*	0.84 (3)	2.03 (3)	2.873 (2)	178 (4)
N2*A*—H2*NA*⋯O1*A*	0.91 (2)	2.17 (2)	2.984 (2)	150 (2)
N3*A*—H4*NA*⋯O4*B*^i^	0.91 (2)	1.97 (3)	2.879 (2)	176 (2)
N3*A*—H5*NA*⋯O1*W*^iii^	0.88 (2)	2.03 (2)	2.908 (2)	172.3 (18)
N1*B*—H1*NB*⋯O3*A*	0.89 (2)	1.92 (2)	2.8077 (19)	175.0 (17)
N2*B*—H2*NB*⋯O4*A*	0.93 (2)	1.98 (2)	2.914 (2)	176.2 (18)
N2*B*—H3*NB*⋯O1*B*^iv^	0.87 (2)	2.34 (2)	3.121 (2)	150.8 (18)
N3*B*—H5*NB*⋯O2*A*^v^	0.92 (3)	2.50 (2)	3.054 (3)	118.8 (19)
N3*B*—H5*NB*⋯O4*A*^v^	0.92 (3)	2.03 (3)	2.938 (3)	168 (2)

Symmetry codes: (i) 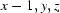; (ii) 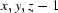; (iii) 
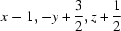
; (iv) 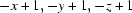; (v) 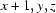.

**Fig. 1.**
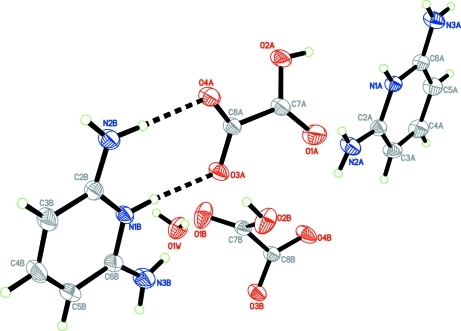
The asymmetric unit of the title compound, showing 30% probability displacement ellipsoids and the atom-numbering scheme.

**Fig. 2.**
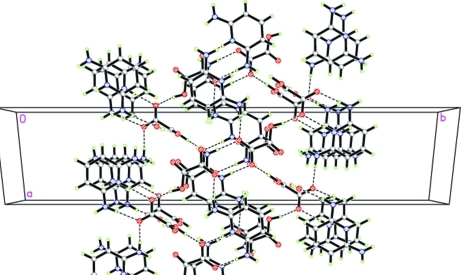
The crystal packing of the title compound (I).

## supplementary crystallographic information

### Comment

2,6-Diaminopyridinium and diaminopyridine in general have an important role in the preparation of aromatic azo dyes, the subject of many polarographic investigations (Abu Zuhri & Cox, 1989).   The crystal structures of 2,6-diaminopyridine (Schwalbe *et al.*, 1987), tetrakis(2,6-diaminopyridinium) diphthalate 2,6-diaminopyridine (Al-Dajani *et al.*, 2009), 2,6-diaminopyridinium pyridinium-2,6-dicarboxylate (Aghabozorg *et al.*, 2005), 2,6-diaminopyridinium hydrogen fumarate (Büyükgüngör & Odabaşoğlu, 2006), bis(2,6-diaminopyridinium) oxalate dihydrate (Odabaşoğlu & Büyükgüngör, 2006), 2,6-diamino pyridinium bromide monohydrate (Haddad & Al-Far, 2003) and 2,6-diamino pyridinium 2-carboxybenzoate (Al-Dajani *et al.*, 2010) have been reported in the literature. Oxalic acid, in principle, exists in three ionization states, viz. singly charged (semioxalate), doubly charged (oxalate) and neutral (oxalic acid).  In order to study some interesting hydrogen bonding interactions, the synthesis and structure of the title compound, (I), is presented here.

The asymmetric unit of the title compound consists of two crystallographically independent 2,6-diaminopyridinium cations (A & B), two hydrogen oxalate anions (A & B) and a water molecule, as shown in Fig. 1.  Each 2,6-diaminopyridinium cation is planar, with a maximum deviation of 0.011 (2)Å for atom C6A in cation A and 0.015 (1)Å for atom N1B in cation B.  In the cations, protonation at atoms N1A and N1B lead to a slight increase in the C2A—N1A—C6A [123.88 (15)°] and C2B—N1B—C6B [123.62 (15)°] angles compared to those observed in an unprotonated structure (Schwalbe *et al.*, 1987). The oxalic acid molecule exists in a mono-ionized state in the crystals. Similar observations were also found in the crystal structure of glycinium hydrogen oxalate (Subha Nandhini *et al.*, 2001), creatininium hydrogen oxalate monohydrate (Bahadur *et al.*, 2007) and 3-carboxypyridinium hydrogen oxalate (Athimoolam & Natarajan, 2007). Here, the hydrogen oxalate anions adopt twisted conformations and the dihedral angles between planes of their carboxylic groups are 31.01 (11)° and 63.48 (11)° for anions A and B, respectively.

In the crystal structure, the carboxylate groups of each hydrogen oxalate anion interact with the corresponding 2,6-diaminopyridinium cations via a pair of N—H···O hydrogen bonds forming an *R*_2_^2^(8) ring motif (Fig. 1) (Bernstein *et al.*, 1995).  The ionic units and water molecules are linked by O—H···O and N—H···O (Table 1) hydrogen bonds to form a three-dimensional network (Fig. 2).

### Experimental

Oxalic acid dihydrate (0.01 mol, 1.3 g) was dissolved in 50 ml of methanol in a round bottom flask. 2,6-diaminopyridine (0.01mol, 1.1 g) was dissolved in 50 ml of methanol in a flask and then added in small portions to the oxalic acid with stirring. The reaction mixture was left stirring for 3 hours at room temperature. Brown precipitate was formed, filtered, and washed with methanol. Recrystallization of the brown precipitate with water has yielded after 48 hours brown crystals which was washed with methanol and dried at 353 K.

### Refinement

The N- and O-bound H atoms were located in a difference map and refined freely [N–H = 0.87 (2)–0.93 (3) Å and O–H = 0.82 (3)– 0.95 (3) Å.  The remaining H atoms were positioned geometrically [C–H = 0.93Å] and were refined using a riding model, with *U*_iso_(H) = 1.2*U*_eq_(C).

### Crystal data

**Table:**

2C_5_H_8_N_3_^+^·2C_2_HO_4_^−^·H_2_O	*F*(000) = 872
*M**_r_* = 416.36	*D*_x_ = 1.484 Mg m^−^^3^
Monoclinic, *P*2_1_/*c*	Mo *K*α radiation, λ = 0.71073 Å
Hall symbol: -P 2ybc	Cell parameters from 8066 reflections
*a* = 8.1681 (1) Å	θ = 2.7–29.6°
*b* = 34.8396 (4) Å	µ = 0.13 mm^−^^1^
*c* = 7.2031 (1) Å	*T* = 296 K
β = 114.573 (1)°	Block, brown
*V* = 1864.16 (4) Å^3^	0.29 × 0.27 × 0.15 mm
*Z* = 4	

### Data collection

**Table:**

Bruker SMART APEXII CCD area-detector diffractometer	4287 independent reflections
Radiation source: fine-focus sealed tube	3195 reflections with *I* > 2σ(*I*)
graphite	*R*_int_ = 0.037
φ and ω scans	θ_max_ = 27.5°, θ_min_ = 2.3°
Absorption correction: multi-scan (*SADABS*; Bruker, 2009)	*h* = −10→10
*T*_min_ = 0.964, *T*_max_ = 0.982	*k* = −45→44
33926 measured reflections	*l* = −9→9

### Refinement

**Table:**

Refinement on *F*^2^	Primary atom site location: structure-invariant direct methods
Least-squares matrix: full	Secondary atom site location: difference Fourier map
*R*[*F*^2^ > 2σ(*F*^2^)] = 0.048	Hydrogen site location: inferred from neighbouring sites
*wR*(*F*^2^) = 0.127	H atoms treated by a mixture of independent and constrained refinement
*S* = 1.03	*w* = 1/[σ^2^(*F*_o_^2^) + (0.0544*P*)^2^ + 0.6267*P*] where *P* = (*F*_o_^2^ + 2*F*_c_^2^)/3
4287 reflections	(Δ/σ)_max_ = 0.001
318 parameters	Δρ_max_ = 0.49 e Å^−^^3^
0 restraints	Δρ_min_ = −0.30 e Å^−^^3^

### Special details

**Table:**

Geometry. All s.u.'s (except the s.u. in the dihedral angle between two l.s. planes) are estimated using the full covariance matrix. The cell s.u.'s are taken into account individually in the estimation of s.u.'s in distances, angles and torsion angles; correlations between s.u.'s in cell parameters are only used when they are defined by crystal symmetry. An approximate (isotropic) treatment of cell s.u.'s is used for estimating s.u.'s involving l.s. planes.
Refinement. Refinement of F^2^ against ALL reflections. The weighted R-factor wR and goodness of fit S are based on F^2^, conventional R-factors R are based on F, with F set to zero for negative F^2^. The threshold expression of F^2^ > 2σ(F^2^) is used only for calculating R-factors(gt) etc. and is not relevant to the choice of reflections for refinement. R-factors based on F^2^ are statistically about twice as large as those based on F, and R- factors based on ALL data will be even larger.

### Fractional atomic coordinates and isotropic or equivalent isotropic displacement parameters (Å^2^)

**Table:**

	*x*	*y*	*z*	*U*_iso_*/*U*_eq_	
O1A	0.4482 (2)	0.61834 (4)	0.9547 (3)	0.0707 (5)	
O2A	0.17712 (17)	0.59774 (4)	0.7486 (2)	0.0604 (4)	
H2A	0.149 (3)	0.6230 (8)	0.768 (4)	0.086 (8)*	
O3A	0.57769 (16)	0.55605 (3)	0.8116 (2)	0.0450 (3)	
O4A	0.32307 (17)	0.52706 (3)	0.7723 (2)	0.0508 (3)	
N1A	0.24244 (19)	0.73699 (4)	0.8214 (2)	0.0379 (3)	
H1NA	0.188 (3)	0.7149 (6)	0.820 (3)	0.055 (6)*	
N2A	0.4866 (3)	0.69987 (5)	0.8523 (3)	0.0532 (4)	
H3NA	0.591 (3)	0.6973 (7)	0.850 (3)	0.067 (7)*	
H2NA	0.431 (3)	0.6779 (7)	0.862 (3)	0.065 (7)*	
N3A	−0.0132 (2)	0.76858 (5)	0.7974 (3)	0.0530 (4)	
H4NA	−0.063 (3)	0.7457 (7)	0.807 (4)	0.074 (7)*	
H5NA	−0.073 (3)	0.7903 (6)	0.783 (3)	0.050 (6)*	
C7A	0.3497 (2)	0.59436 (5)	0.8417 (3)	0.0426 (4)	
C8A	0.4205 (2)	0.55548 (5)	0.8045 (3)	0.0366 (4)	
C2A	0.4143 (2)	0.73481 (5)	0.8371 (3)	0.0395 (4)	
C3A	0.5024 (3)	0.76876 (6)	0.8363 (3)	0.0486 (4)	
H3A	0.6205	0.7684	0.8493	0.058*	
C4A	0.4130 (3)	0.80282 (6)	0.8162 (3)	0.0547 (5)	
H4A	0.4714	0.8255	0.8127	0.066*	
C5A	0.2398 (3)	0.80454 (5)	0.8011 (3)	0.0516 (5)	
H5A	0.1820	0.8280	0.7876	0.062*	
C6A	0.1525 (2)	0.77058 (5)	0.8062 (3)	0.0400 (4)	
O3B	1.06013 (16)	0.66502 (3)	0.7787 (2)	0.0479 (3)	
O4B	0.83371 (18)	0.69483 (4)	0.8123 (2)	0.0584 (4)	
O1B	0.7509 (2)	0.61351 (4)	0.5650 (2)	0.0694 (5)	
O2B	0.7631 (2)	0.61662 (5)	0.8760 (2)	0.0667 (5)	
H2B	0.690 (4)	0.5942 (9)	0.840 (4)	0.104 (9)*	
N1B	0.72190 (18)	0.48805 (4)	0.7325 (2)	0.0376 (3)	
H1NB	0.671 (3)	0.5096 (6)	0.750 (3)	0.049 (5)*	
N2B	0.4688 (2)	0.45485 (5)	0.7030 (3)	0.0493 (4)	
H2NB	0.423 (3)	0.4775 (7)	0.731 (3)	0.062 (6)*	
H3NB	0.408 (3)	0.4336 (7)	0.672 (3)	0.073 (7)*	
N3B	0.9553 (3)	0.52718 (5)	0.7489 (3)	0.0582 (5)	
H5NB	1.071 (3)	0.5308 (7)	0.762 (3)	0.069 (7)*	
H4NB	0.888 (4)	0.5452 (7)	0.739 (4)	0.076 (8)*	
C8B	0.9063 (2)	0.66636 (5)	0.7784 (3)	0.0371 (4)	
C7B	0.7974 (2)	0.62902 (5)	0.7277 (3)	0.0376 (4)	
C2B	0.6357 (2)	0.45385 (5)	0.7121 (3)	0.0383 (4)	
C3B	0.7258 (3)	0.42076 (5)	0.7024 (3)	0.0498 (5)	
H3B	0.6709	0.3969	0.6882	0.060*	
C4B	0.8978 (3)	0.42379 (6)	0.7143 (3)	0.0535 (5)	
H4B	0.9594	0.4015	0.7108	0.064*	
C5B	0.9815 (3)	0.45863 (6)	0.7310 (3)	0.0497 (5)	
H5B	1.0976	0.4600	0.7379	0.060*	
C6B	0.8898 (2)	0.49165 (5)	0.7376 (3)	0.0415 (4)	
O1W	0.8207 (2)	0.65644 (5)	0.2651 (3)	0.0634 (4)	
H2W	0.798 (4)	0.6437 (8)	0.351 (4)	0.096 (10)*	
H1W	0.759 (4)	0.6459 (9)	0.157 (5)	0.098 (10)*	

### Atomic displacement parameters (Å^2^)

**Table:**

	*U*^11^	*U*^22^	*U*^33^	*U*^12^	*U*^13^	*U*^23^
O1A	0.0486 (9)	0.0399 (8)	0.1120 (13)	−0.0056 (6)	0.0219 (8)	−0.0224 (8)
O2A	0.0372 (8)	0.0412 (8)	0.1012 (11)	0.0008 (6)	0.0271 (7)	−0.0223 (7)
O3A	0.0376 (7)	0.0333 (6)	0.0729 (8)	−0.0022 (5)	0.0317 (6)	−0.0039 (6)
O4A	0.0405 (7)	0.0302 (6)	0.0870 (9)	−0.0055 (5)	0.0319 (7)	−0.0086 (6)
N1A	0.0361 (8)	0.0298 (7)	0.0486 (8)	−0.0047 (6)	0.0185 (6)	0.0009 (6)
N2A	0.0470 (10)	0.0429 (10)	0.0781 (12)	0.0037 (8)	0.0344 (9)	0.0017 (8)
N3A	0.0452 (10)	0.0352 (9)	0.0845 (12)	0.0038 (8)	0.0329 (9)	0.0058 (8)
C7A	0.0373 (10)	0.0314 (9)	0.0640 (11)	−0.0022 (7)	0.0259 (8)	−0.0030 (8)
C8A	0.0339 (9)	0.0300 (8)	0.0489 (9)	−0.0025 (7)	0.0203 (7)	−0.0012 (7)
C2A	0.0387 (9)	0.0389 (9)	0.0416 (8)	−0.0012 (7)	0.0172 (7)	−0.0007 (7)
C3A	0.0376 (10)	0.0504 (11)	0.0600 (11)	−0.0101 (8)	0.0223 (8)	−0.0011 (8)
C4A	0.0553 (12)	0.0384 (10)	0.0720 (13)	−0.0147 (9)	0.0279 (10)	−0.0001 (9)
C5A	0.0525 (12)	0.0306 (9)	0.0719 (12)	−0.0024 (8)	0.0261 (10)	0.0029 (8)
C6A	0.0409 (9)	0.0331 (9)	0.0463 (9)	−0.0012 (7)	0.0186 (7)	0.0014 (7)
O3B	0.0367 (7)	0.0328 (6)	0.0814 (9)	−0.0065 (5)	0.0317 (6)	−0.0092 (6)
O4B	0.0447 (8)	0.0325 (7)	0.1075 (11)	−0.0017 (6)	0.0412 (8)	−0.0090 (7)
O1B	0.0931 (12)	0.0613 (9)	0.0681 (9)	−0.0413 (8)	0.0479 (9)	−0.0255 (7)
O2B	0.0829 (11)	0.0623 (10)	0.0626 (9)	−0.0404 (8)	0.0379 (8)	−0.0107 (7)
N1B	0.0342 (8)	0.0293 (7)	0.0525 (8)	0.0011 (6)	0.0213 (6)	−0.0033 (6)
N2B	0.0404 (9)	0.0338 (8)	0.0799 (11)	−0.0065 (7)	0.0314 (8)	−0.0075 (8)
N3B	0.0431 (10)	0.0469 (10)	0.0936 (14)	−0.0114 (8)	0.0374 (10)	−0.0109 (9)
C8B	0.0321 (9)	0.0299 (8)	0.0500 (9)	−0.0009 (7)	0.0179 (7)	−0.0007 (7)
C7B	0.0289 (8)	0.0316 (8)	0.0542 (10)	−0.0011 (7)	0.0191 (7)	−0.0022 (7)
C2B	0.0387 (9)	0.0327 (9)	0.0454 (9)	−0.0015 (7)	0.0194 (7)	−0.0007 (7)
C3B	0.0538 (12)	0.0287 (9)	0.0701 (12)	0.0037 (8)	0.0288 (10)	0.0008 (8)
C4B	0.0526 (12)	0.0430 (11)	0.0678 (12)	0.0168 (9)	0.0279 (10)	0.0014 (9)
C5B	0.0353 (10)	0.0554 (12)	0.0610 (11)	0.0068 (8)	0.0226 (8)	−0.0024 (9)
C6B	0.0348 (9)	0.0430 (10)	0.0487 (9)	−0.0026 (7)	0.0194 (7)	−0.0052 (7)
O1W	0.0745 (11)	0.0547 (9)	0.0603 (10)	−0.0163 (8)	0.0274 (9)	−0.0031 (8)

### Geometric parameters (Å, °)

**Table:**

O1A—C7A	1.209 (2)	O4B—C8B	1.231 (2)
O2A—C7A	1.290 (2)	O1B—C7B	1.200 (2)
O2A—H2A	0.94 (3)	O2B—C7B	1.286 (2)
O3A—C8A	1.2637 (19)	O2B—H2B	0.95 (3)
O4A—C8A	1.2303 (19)	N1B—C2B	1.360 (2)
N1A—C6A	1.362 (2)	N1B—C6B	1.362 (2)
N1A—C2A	1.362 (2)	N1B—H1NB	0.89 (2)
N1A—H1NA	0.89 (2)	N2B—C2B	1.338 (2)
N2A—C2A	1.337 (2)	N2B—H2NB	0.93 (2)
N2A—H3NA	0.87 (2)	N2B—H3NB	0.87 (3)
N2A—H2NA	0.91 (2)	N3B—C6B	1.338 (2)
N3A—C6A	1.330 (2)	N3B—H5NB	0.92 (3)
N3A—H4NA	0.91 (3)	N3B—H4NB	0.82 (3)
N3A—H5NA	0.88 (2)	C8B—C7B	1.532 (2)
C7A—C8A	1.539 (2)	C2B—C3B	1.385 (2)
C2A—C3A	1.386 (2)	C3B—C4B	1.376 (3)
C3A—C4A	1.369 (3)	C3B—H3B	0.9300
C3A—H3A	0.9300	C4B—C5B	1.374 (3)
C4A—C5A	1.374 (3)	C4B—H4B	0.9300
C4A—H4A	0.9300	C5B—C6B	1.385 (3)
C5A—C6A	1.390 (2)	C5B—H5B	0.9300
C5A—H5A	0.9300	O1W—H2W	0.84 (3)
O3B—C8B	1.2567 (19)	O1W—H1W	0.82 (3)
			
C7A—O2A—H2A	106.9 (16)	C2B—N1B—C6B	123.62 (15)
C6A—N1A—C2A	123.88 (15)	C2B—N1B—H1NB	120.2 (13)
C6A—N1A—H1NA	119.4 (14)	C6B—N1B—H1NB	116.1 (13)
C2A—N1A—H1NA	116.8 (14)	C2B—N2B—H2NB	120.2 (14)
C2A—N2A—H3NA	119.8 (16)	C2B—N2B—H3NB	117.0 (16)
C2A—N2A—H2NA	123.8 (14)	H2NB—N2B—H3NB	123 (2)
H3NA—N2A—H2NA	116 (2)	C6B—N3B—H5NB	120.1 (15)
C6A—N3A—H4NA	121.2 (16)	C6B—N3B—H4NB	117.6 (19)
C6A—N3A—H5NA	117.9 (13)	H5NB—N3B—H4NB	122 (2)
H4NA—N3A—H5NA	121 (2)	O4B—C8B—O3B	126.38 (15)
O1A—C7A—O2A	124.31 (16)	O4B—C8B—C7B	116.86 (14)
O1A—C7A—C8A	122.17 (16)	O3B—C8B—C7B	116.75 (14)
O2A—C7A—C8A	113.46 (15)	O1B—C7B—O2B	124.77 (16)
O4A—C8A—O3A	125.94 (15)	O1B—C7B—C8B	122.12 (16)
O4A—C8A—C7A	118.73 (14)	O2B—C7B—C8B	113.11 (15)
O3A—C8A—C7A	115.33 (14)	N2B—C2B—N1B	117.00 (15)
N2A—C2A—N1A	117.55 (16)	N2B—C2B—C3B	124.81 (17)
N2A—C2A—C3A	124.36 (17)	N1B—C2B—C3B	118.19 (16)
N1A—C2A—C3A	118.09 (16)	C4B—C3B—C2B	118.91 (17)
C4A—C3A—C2A	119.02 (17)	C4B—C3B—H3B	120.5
C4A—C3A—H3A	120.5	C2B—C3B—H3B	120.5
C2A—C3A—H3A	120.5	C5B—C4B—C3B	122.08 (17)
C3A—C4A—C5A	122.15 (18)	C5B—C4B—H4B	119.0
C3A—C4A—H4A	118.9	C3B—C4B—H4B	119.0
C5A—C4A—H4A	118.9	C4B—C5B—C6B	118.73 (17)
C4A—C5A—C6A	118.93 (18)	C4B—C5B—H5B	120.6
C4A—C5A—H5A	120.5	C6B—C5B—H5B	120.6
C6A—C5A—H5A	120.5	N3B—C6B—N1B	117.38 (17)
N3A—C6A—N1A	117.60 (16)	N3B—C6B—C5B	124.22 (17)
N3A—C6A—C5A	124.52 (17)	N1B—C6B—C5B	118.40 (16)
N1A—C6A—C5A	117.88 (16)	H2W—O1W—H1W	103 (3)
C7B—O2B—H2B	112.3 (18)		
			
O1A—C7A—C8A—O4A	147.14 (19)	O4B—C8B—C7B—O1B	116.2 (2)
O2A—C7A—C8A—O4A	−29.9 (2)	O3B—C8B—C7B—O1B	−62.9 (2)
O1A—C7A—C8A—O3A	−32.5 (3)	O4B—C8B—C7B—O2B	−64.0 (2)
O2A—C7A—C8A—O3A	150.49 (17)	O3B—C8B—C7B—O2B	116.95 (18)
C6A—N1A—C2A—N2A	179.59 (16)	C6B—N1B—C2B—N2B	−177.96 (16)
C6A—N1A—C2A—C3A	−0.4 (2)	C6B—N1B—C2B—C3B	2.1 (3)
N2A—C2A—C3A—C4A	178.82 (19)	N2B—C2B—C3B—C4B	−179.77 (18)
N1A—C2A—C3A—C4A	−1.2 (3)	N1B—C2B—C3B—C4B	0.2 (3)
C2A—C3A—C4A—C5A	1.4 (3)	C2B—C3B—C4B—C5B	−1.4 (3)
C3A—C4A—C5A—C6A	0.0 (3)	C3B—C4B—C5B—C6B	0.5 (3)
C2A—N1A—C6A—N3A	−178.21 (16)	C2B—N1B—C6B—N3B	177.04 (17)
C2A—N1A—C6A—C5A	1.8 (3)	C2B—N1B—C6B—C5B	−3.0 (3)
C4A—C5A—C6A—N3A	178.47 (19)	C4B—C5B—C6B—N3B	−178.40 (19)
C4A—C5A—C6A—N1A	−1.5 (3)	C4B—C5B—C6B—N1B	1.7 (3)

### Hydrogen-bond geometry (Å, °)

**Table:**

*D*—H···*A*	*D*—H	H···*A*	*D*···*A*	*D*—H···*A*
N1A—H1NA···O3B^i^	0.89 (2)	1.99 (2)	2.8668 (19)	172.5 (18)
O1W—H1W···O1A^ii^	0.82 (3)	2.53 (3)	3.219 (3)	142 (3)
O1W—H1W···O2B^ii^	0.82 (3)	2.28 (3)	2.984 (2)	145 (3)
N2A—H3NA···O4B	0.86 (3)	2.11 (3)	2.972 (3)	173.3 (18)
O2A—H2A···O3B^i^	0.94 (3)	1.65 (3)	2.5743 (18)	169 (3)
O2B—H2B···O3A	0.95 (3)	1.58 (3)	2.525 (2)	172 (3)
O1W—H2W···O1B	0.84 (3)	2.03 (3)	2.873 (2)	178 (4)
N2A—H2NA···O1A	0.91 (2)	2.17 (2)	2.984 (2)	150 (2)
N3A—H4NA···O4B^i^	0.91 (2)	1.97 (3)	2.879 (2)	176 (2)
N3A—H5NA···O1W^iii^	0.88 (2)	2.03 (2)	2.908 (2)	172.3 (18)
N1B—H1NB···O3A	0.89 (2)	1.92 (2)	2.8077 (19)	175.0 (17)
N2B—H2NB···O4A	0.93 (2)	1.98 (2)	2.914 (2)	176.2 (18)
N2B—H3NB···O1B^iv^	0.87 (2)	2.34 (2)	3.121 (2)	150.8 (18)
N3B—H5NB···O2A^v^	0.92 (3)	2.50 (2)	3.054 (3)	118.8 (19)
N3B—H5NB···O4A^v^	0.92 (3)	2.03 (3)	2.938 (3)	168 (2)

Symmetry codes: (i) *x*−1, *y*, *z*; (ii) *x*, *y*, *z*−1; (iii) *x*−1, −*y*+3/2, *z*+1/2; (iv) −*x*+1, −*y*+1, −*z*+1; (v) *x*+1, *y*, *z*.

## References

[bb1] Abu Zuhri, A. Z. & Cox, J. A. (1989). *Mikrochim. Acta*, **11**, 277–283.

[bb2] Aghabozorg, H., Akbari Saei, A. & Ramezanipour, F. (2005). *Acta Cryst.* E**61**, o3242–o3244.

[bb3] Al-Dajani, M. T. M., Abdallah, H. H., Mohamed, N., Rosli, M. M. & Fun, H.-K. (2010). *Acta Cryst.* E**66**, o2433–o2434. 10.1107/S1600536810032903 PMC3008098 21588757

[bb4] Al-Dajani, M. T. M., Salhin, A., Mohamed, N., Loh, W.-S. & Fun, H.-K. (2009). *Acta Cryst.* E**65**, o2931–o2932. 10.1107/S1600536809044468 PMC2971263 21578508

[bb5] Athimoolam, S. & Natarajan, S. (2007). *Acta Cryst.* E**63**, o963–o965.

[bb6] Bahadur, S. A., Kannan, R. S. & Sridhar, B. (2007). *Acta Cryst.* E**63**, o2387–o2389.

[bb7] Bernstein, J., Davis, R. E., Shimoni, L. & Chang, N.-L. (1995). *Angew. Chem. Int. Ed. Engl.* **34**, 1555–1573.

[bb8] Bruker (2009). *APEX2*, *SAINT* and *SADABS* Bruker AXS Inc., Madison, Wisconsin, USA.

[bb9] Büyükgüngör, O. & Odabąsoǧlu, M. (2006). *Acta Cryst.* E**62**, o3816–o3818.

[bb10] Haddad, S. F. & Al-Far, R. H. (2003). *Acta Cryst.* E**59**, o1444–o1446.

[bb12] Odabaşoğlu, M. & Büyükgüngör, O. (2006). *Acta Cryst.* E**62**, o4543–o4544.

[bb13] Schwalbe, C. H., Williams, G. J. B. & Koetzle, T. F. (1987). *Acta Cryst.* C**43**, 2191–2195.

[bb14] Sheldrick, G. M. (2008). *Acta Cryst.* A**64**, 112–122. 10.1107/S0108767307043930 18156677

[bb15] Spek, A. L. (2009). *Acta Cryst.* D**65**, 148–155. 10.1107/S090744490804362X PMC2631630 19171970

[bb11] Subha Nandhini, M., Krishnakumar, R. V. & Natarajan, S. (2001). *Acta Cryst.* C**57**, 115–116. 10.1107/s0108270100015456 11173420

